# IG and TR single chain fragment variable (scFv) sequence analysis: a new advanced functionality of IMGT/V-QUEST and IMGT/HighV-QUEST

**DOI:** 10.1186/s12865-017-0218-8

**Published:** 2017-06-26

**Authors:** Véronique Giudicelli, Patrice Duroux, Sofia Kossida, Marie-Paule Lefranc

**Affiliations:** IMGT®, the international ImMunoGeneTics information system®, Laboratoire d’ImmunoGénétique Moléculaire LIGM, Institut de Génétique Humaine IGH, UMR 9002, CNRS, Montpellier University, Montpellier, France

**Keywords:** IMGT, immunoglobulin, IG, T cell receptor, TR, single chain fragment variable, scFv, IMGT-ONTOLOGY, V-DOMAIN, adaptive immune repertoire

## Abstract

**Background:**

IMGT®, the international ImMunoGeneTics information system® (http://www.imgt.org), was created in 1989 in Montpellier, France (CNRS and Montpellier University) to manage the huge and complex diversity of the antigen receptors, and is at the origin of immunoinformatics, a science at the interface between immunogenetics and bioinformatics. Immunoglobulins (IG) or antibodies and T cell receptors (TR) are managed and described in the IMGT® databases and tools at the level of receptor, chain and domain. The analysis of the IG and TR variable (V) domain rearranged nucleotide sequences is performed by IMGT/V-QUEST (online since 1997, 50 sequences per batch) and, for next generation sequencing (NGS), by IMGT/HighV-QUEST, the high throughput version of IMGT/V-QUEST (portal begun in 2010, 500,000 sequences per batch). In vitro combinatorial libraries of engineered antibody single chain Fragment variable (scFv) which mimic the in vivo natural diversity of the immune adaptive responses are extensively screened for the discovery of novel antigen binding specificities. However the analysis of NGS full length scFv (~850 bp) represents a challenge as they contain two V domains connected by a linker and there is no tool for the analysis of two V domains in a single chain.

**Methods:**

The functionality "Analyis of single chain Fragment variable (scFv)" has been implemented in IMGT/V-QUEST and, for NGS, in IMGT/HighV-QUEST for the analysis of the two V domains of IG and TR scFv. It proceeds in five steps: search for a first closest V-REGION, full characterization of the first V-(D)-J-REGION, then search for a second V-REGION and full characterization of the second V-(D)-J-REGION, and finally linker delimitation.

**Results:**

For each sequence or NGS read, positions of the 5′V-DOMAIN, linker and 3′V-DOMAIN in the scFv are provided in the ‘V-orientated’ sense. Each V-DOMAIN is fully characterized (gene identification, sequence description, junction analysis, characterization of mutations and amino changes). The functionality is generic and can analyse any IG or TR single chain nucleotide sequence containing two V domains, provided that the corresponding species IMGT reference directory is available.

**Conclusion:**

The “Analysis of single chain Fragment variable (scFv)” implemented in IMGT/V-QUEST and, for NGS, in IMGT/HighV-QUEST provides the identification and full characterization of the two V domains of full-length scFv (~850 bp) nucleotide sequences from combinatorial libraries. The analysis can also be performed on concatenated paired chains of expressed antigen receptor IG or TR repertoires.

## Background

The efficiency of the adaptive immune responses of humans and other jawed vertebrates (or *gnathostomata*) results from the remarkable immune specificity and memory, which are the properties of B and T cells owing to an extreme diversity of their antigen receptors [1]. The specific antigen receptors comprise the immunoglobulins (IG) or antibodies [2], expressed on the surface of the B cells and secreted by the plasmocytes, and the T cell receptors (TR) [3] expressed on the surface of T cells. The potential antigen receptor repertoire of each individual is estimated to comprise about 2 x 10^12^ different IG and TR specificities, and the limiting factor is the number of B and T cells that an organism is genetically programmed to produce [1].

IMGT®, the international ImMunoGeneTics information system® [4, 5], was created in 1989 by Marie-Paule Lefranc at Montpellier, France (CNRS and Montpellier University) to manage the huge and complex diversity of these antigen receptors, and is at the origin of immunoinformatics, a science at the interface between immunogenetics and bioinformatics [1]. IMGT® has developed IMGT-ONTOLOGY [6] to manage, reuse and share knowledge in immunoinformatics [1]. IMGT-ONTOLOGY comprises seven axioms which generated the concepts of identification, description, classification, numerotation, localization, orientation and obtention and the IMGT Scientific chart rules (keywords, labels, numbering): IDENTIFICATION (IMGT® standardized keywords) [7], DESCRIPTION (IMGT® standardized labels (in capital letters, no plural)) [8], CLASSIFICATION (IMGT® standardized gene and allele nomenclature) [9], NUMEROTATION (IMGT unique numbering [10–12] and its graphical 2D representation or IMGT Collier de Perles [13]) [14–16], LOCALIZATION, ORIENTATION and OBTENTION [17–19].

IMGT® is specialized in the IG or antibodies, TR, major histocompatibility (MH) of human and other jawed vertebrate species, and in the immunoglobulin superfamily (IgSF), MH superfamily (MhSF) and related proteins of the immune system (RPI) of vertebrates and invertebrates. IMGT® comprises 7 databases, seventeen online tools and more than 20,000 pages of Web resources, available at the IMGT® Home page [4, 5]. The databases provide IMGT biocurated and standardized information on genes (IMGT/GENE-DB [20], sequences (IMGT/LIGM-DB [21], IMGT/PRIMER-DB), two-dimensional (2D) and three-dimensional (3D) structures (IMGT/2Dstructure-DB and IMGT/3Dstructure-DB [22, 23]), therapeutic monoclonal antibodies, fusion proteins for immune applications (FPIA), composite proteins for clinical applications (CPCA) and related proteins of the immune system (RPI) (IMGT/mAb-DB [4]). The online tools are available for the analysis of nucleotide sequences (IMGT/V-QUEST [24–26], IMGT/JunctionAnalysis [27, 28], IMGT/Automat [29, 30]), next generation sequencing (NGS) nucleotide sequences (IMGT/HighV-QUEST [31–35]), amino acid sequences (IMGT/DomainGapAlign [36], IMGT/Collier-de-Perles [37]), genes (IMGT/GeneInfo [38], IMGT/LIGMotif [39], IMGT/GeneFrequency) and 2D and 3D structures (IMGT/StructuralQuery). The standalone tool, IMGT/StatClonotype [40, 41], allows statistical comparison of clonotype diversity and expression from IMGT/HighV-QUEST NGS results.

IG and TR are managed and described in the IMGT® databases and tools at the level of receptor, chain and domain [1]. A complete IgG1 is made of 12 domains belonging to two identical heavy (H) chains (4 domains each) and two identical light (L) chains (2 domains each) [1]. The N-terminal domain of each IG H and L chain is a variable domain (VH and VL, respectively) which results from the rearrangement at the DNA level of three genes for the VH (variable (V), diversity (D) and joining (J)) and of two genes for the VL (V and J). As a result a VH is encoded by a V-D-J-REGION whereas a VL is encoded by a V-J-REGION (Table 1) [2]. Similarly the N-terminal domain of each chain of a T cell receptor (TR) is a variable domain encoded by a V-D-J-REGION or a V-J-REGION (Table 1) [3].

**Table 1:** V-DOMAIN types analyzed by IMGT/V-QUEST

Receptor type	V-DOMAIN description			Locus name	Chain type (transcript or protein)
	Structure labels (IMGT/3Dstructure-DB)		Sequence labels (IMGT/LIGM-DB)		
IG	VH		V-D-J-REGION	IGH	IG-Heavy
	VL^a^	V-KAPPA	V-J-REGION	IGK	IG-Light-Kappa
		V-LAMBDA	V-J-REGION	IGL	IG-Light-Lambda
		V-IOTA^b^	V-J-REGION	IGI	IG-Light-Iota
TR	V-ALPHA		V-J-REGION	TRA	TR-Alpha
	V-BETA		V-D-J-REGION	TRB	TR-Beta
	V-GAMMA		V-J-REGION	TRG	TR-Gamma
	V-DELTA		V-D-J-REGION	TRD	TR-Delta

^a^V-RHO of the IG-Light-Rho (frog) and V-SIGMA of the Ig-Light-Sigma (Chondrichthyes and frog) are not shown but will be analyzed once the genomic germline V and J genes are sequenced and available in the IMGT/V-QUEST reference directory (Correspondence between chain types and C genes: IG and TR (all vertebrate species) [60])

^b^V-IOTA is the VL of the IG-Light-Iota chain type (transcript or protein) of the Chondrichthyes and Actinopterygii (which include the Teleostei)

The analysis of the IG and TR V domain rearranged nucleotide sequences is performed by IMGT/V-QUEST (online since 1997, 50 sequences per batch) and, for NGS, by IMGT/HighV-QUEST, the high throughput version of IMGT/V-QUEST (online since October 2010), maximum of 500,000 sequences per batch, set comparison of 1 million results). IMGT/V-QUEST and HighV-QUEST use the same algorithm and the same IMGT reference directories [4].

**Fig. 1:**
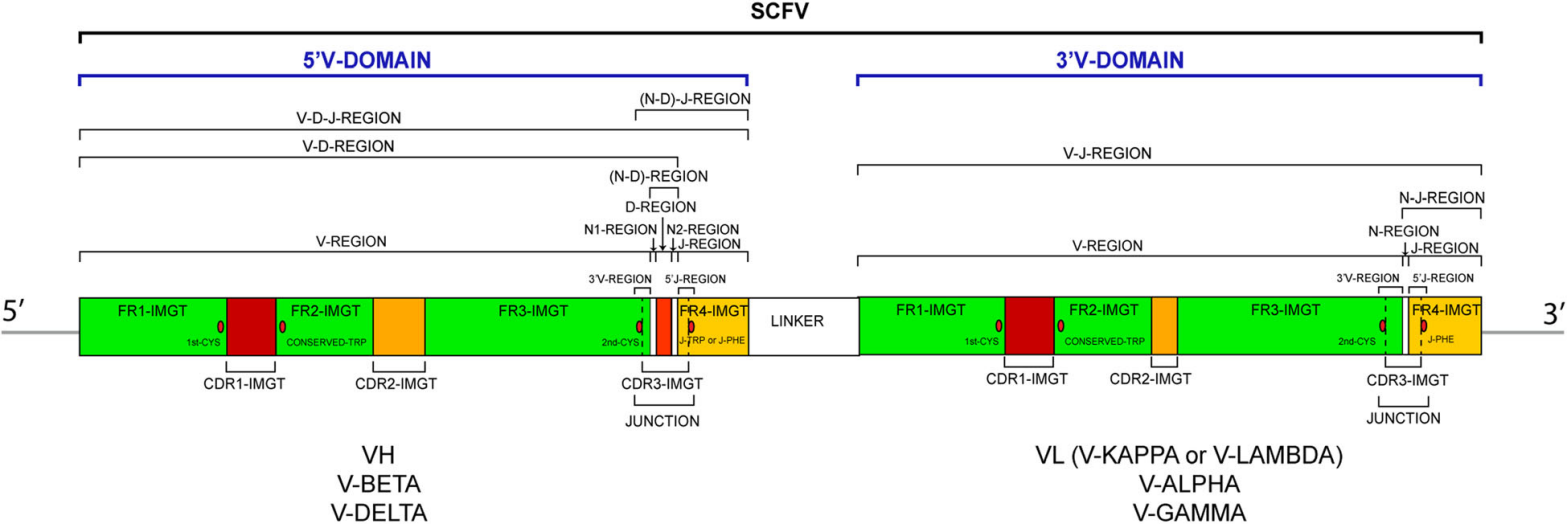
Representation of a scFv with the IMGT Labels for the two V-DOMAIN. The relative position 5′ and 3′ of the two V-DOMAIN in the scFv, in this figure, is arbitrary. At least 23 and 19 labels are necessary to describe a V-D-J-REGION and a V-J-REGION, respectively in IMGT/V-QUEST [59]. The conserved 1st-CYS 23, CONSERVED-TRP 41, 2nd-CYS 104 and J-TRP or J-PHE 118 based on the IMGT unique numbering are shown in both V-DOMAIN [1, 10, 14, 16]

So far, the analysis has been performed on each V domain individually. The Sanger sequencing of single chain Fragment variable (scFv) was done on a case by case basis using IMGT/V-QUEST. Indeed scFv are single chains of approximate molecular weight of 26,000 Da, encoded by about 800–900 nucleotides with two V domains connected by a linker of about 45-60 nucleotides (Fig. 1), and the user could easily identify the linker by its sequence and length (for example (GSSS)3) and remove it or split the sequence in two parts preceding IMGT/V-QUEST analysis. This manual approach is cumbersome and not applicable to high-throughput sequencing. The NGS sequencing of scFv from combinatorial libraries has been limited up to now by the short length of reads, however with the availability of longer NGS reads (1000 bp and more) and the use of circular consensus sequencing (CCS) [42] as introduced by Pacific Biosciences, high quality sequencing of full-length scFv or of single cell concatenated antigen receptor V-domain or chain pairs are expected.

In this paper, we describe a new advanced IMGT/V-QUEST functionality “Analysis of single chain Fragment variable (scFv)” for the identification and characterization of the two variable domains of scFv, generic for IG and TR, and implemented, for NGS, in IMGT/HighV-QUEST.

## Methods

**Fig. 2:**
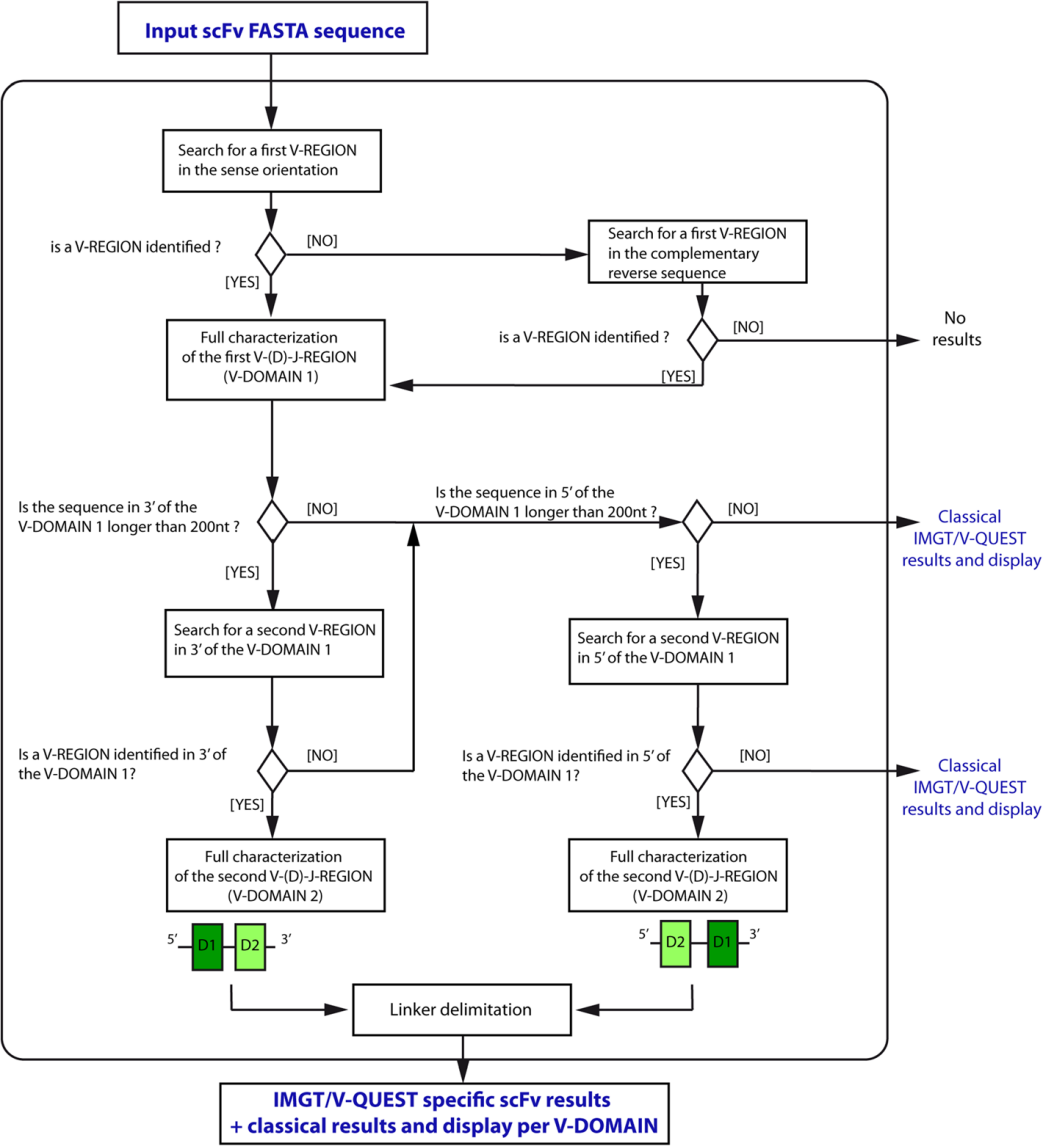
Main steps of IMGT/V-QUEST algorithm for the analysis of scFv sequences. ‘D1’: indicates the first identified and characterized V-(D)-J-REGION (V-DOMAIN 1). ‘D2’: indicates the second identified and characterized V-(D)-J-REGION (V-DOMAIN 2) which can be found in 3′ or in 5′ of ‘D1’ in the ‘V-orientated’ sequence

The algorithm proceeds in five steps (Fig. 2): search for a first closest V-REGION, full characterization of the first V-(D)-J-REGION, then search for a second V-REGION and full characterization of the second V-(D)-J-REGION, and finally linker delimitation.

### Search for a first closest V-REGION

For a selected species and receptor type (IG or TR), the IMGT/V-QUEST tool first searches the submitted sequence for the closest V-REGION by comparison with the IMGT reference directory of the V groups of the selected receptor type (for the IG: IGHV, IGKV and IGLV; for the TR: TRAV, TRBV, TRGV and TRDV) [26]. The IMGT reference directories [4] are reference sequences of IG and TR IMGT genes and alleles (functional (F), open reading frames (ORF) and in-frame pseudogenes (P)), from IMGT/GENE-DB [20]. By default, the search is done on ‘F + ORF + in-frame P’. The identification of the closest V-REGION determines the assignment of the genes of the V-(D)-J-REGION to a locus (IGH, IGK or IGL for IG, or TRA, TRB, TRG or TRD for TR, respectively).

The first closest V-REGION identified is the one with the highest score which would have been detected in a classical IMGT/V-QUEST analysis (i.e., without the option “Analysis of single chain Fragment variable (scFv)”). There is no search priority for a given V group or for a respective order position (5′ or 3′) in the submitted sequence.

If no V-REGION is identified, IMGT/V-QUEST complementary reverses the submitted (input) sequence automatically, and the search is performed again. If a closest V-REGION is identified, this defines the complementary reverse sequence as being in the ‘sense’ orientation for the V-REGION (Fig. 2).

The following steps of the algorithm are performed on scFv sequences in which the V-REGION has a ‘sense’ orientation, and therefore are designated as ‘V-orientated scFv’ (either from the direct input scFv sequence or as a result of a complementary reverse step).

### Full characterization of the first V-(D)-J-REGION

The full characterization of the first identified V-(D)-J-REGION (‘V-DOMAIN 1’ or ‘D1’ in Fig. 2) is performed through a set of methods described previously [24–26]. In summary, IMGT/V-QUEST

i. identifies the names of the closest germline V-GENE and allele and J-GENE and allele, with score and percent (%) of identity [24–26] by alignments with the IMGT reference directory [4].
ii. adds gaps according to the IMGT unique numbering [10] and determines the lengths of the four framework regions (FR) FR1-IMGT to FR4-IMGT, and those of the three complementarity determining regions (CDR), CDR1-IMGT to CDR3-IMGT [1].
iii. delimits the V-(D)-J-REGION, i.e., the V-DOMAIN (V-D-J-REGION for the IGH, TRB and TRD loci or V-J-REGION for the IGK, IGL, TRA and TRG loci),
iv. provides a detailed analysis of the V-(D)-J junction and the identification of the D genes and alleles for IGH, TRB and TRD performed by the integrated IMGT/JunctionAnalysis tool [27, 28],
v. provides an extensive analysis of the nucleotide (nt) mutations and amino acid (AA) changes, resulting for the IG from somatic hypermutations, by comparison with the closest V-REGION,
vi. localizes the mutation hotspots in the closest germline V gene and allele,
vii. and finally, annotates the V-(D)-J-REGION identified with IMGT labels using IMGT/Automat [29, 30].

Links to the IMGT/Collier-de-Perles tool graphical representation [34] are only incorporated for IMGT/V-QUEST results online.

### Search for a second V-REGION and full characterization of the second V-(D)-J-REGION

Following the complete characterization of a first V-(D)-J-REGION (‘V-DOMAIN 1’ or ‘D1’ in Fig. 2), a second V-REGION is searched by comparison with the V groups of the same receptor type (IG or TR) and species as previously selected, from the IMGT reference directory. The search is performed on a ‘V-orientated’ sequence, which is either the input sequence if the ‘D1’ has a ‘sense’ orientation or the complementary reverse sequence if the ‘D1’ has an “antisense” orientation.

The search is performed first between the 3′end of ‘D1’ and the 3′end of the V-orientated sequence, provided that this part has at least a length of 200 nt (Fig. 2). If a V-REGION is identified, the full characterization of the second V-(D)-J-REGION (‘V-DOMAIN 2′ or ‘D2’ in Fig. 2) is performed by IMGT/V-QUEST similarly to that of the first V-(D)-J-REGION, as described above [24–30] (Fig. 2).

If IMGT/V-QUEST does not find a second V-REGION in 3′ of the V-orientated sequence (either sequence shorter than 200 nt or absence of results), a similar search is performed between the 5′ end of the V-orientated sequence and the 5′ end of ‘D1’, provided that this part of the sequence has at least a length of 200 nt. If a V-REGION is identified, the full characterization of the second V-(D)-J-REGION (‘V-DOMAIN 2′ or ‘D2’ in Fig. 2) is performed by IMGT/V-QUEST similarly to that of the first V-(D)-J-REGION, as described above [24–30] (Fig. 2).

#### Linker delimitation

When two V-(D)-J-REGION (‘V-DOMAIN 1’ and ‘V-DOMAIN 2’) are characterized, the sequence between them is delimited and defined as ‘linker’ (Fig. 2). The linker length and positions in the sequence are delimited by the 3′ end of ‘V-DOMAIN 1′ and the 5′ end of ‘V-DOMAIN 2’. There is no further characterization of the linker sequence.

## Results

### IMGT/V-QUEST user submission for “Analysis of single chain Fragment variable (scFv)”

The IMGT/V-QUEST novel functionality for “Analysis of single chain Fragment variable (scFv)” is freely available online for academics (since May 10, 2016). The scFv sequences are submitted in FASTA format (up to 50 sequences, or a maximum of 10 sequences with the option ‘Search for insertions and deletions’).

**Fig. 3:**
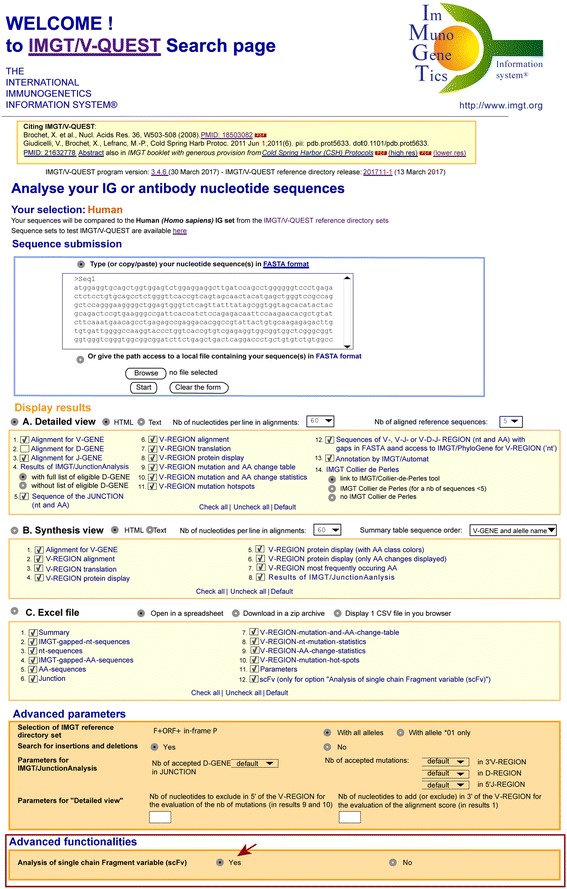
IMGT/V-QUEST Search page. The analysis of scFv is an option of IMGT/V-QUEST available in the section Advanced functionalities at the bottom of the IMGT/V-QUEST Search page. This option is not selected by default

The “Analysis of single chain Fragment variable (scFv)” is selected as an option in “Advanced functionalities” at the bottom of the IMGT/V-QUEST Search page (Fig. 3).

The user can choose one of the three displays: A. Detailed view. B. Synthesis view. C. Excel file. Selecting the “Analysis of single chain Fragment variable (scFv)” adds automatically the file ‘12’ in the display C. Excel file.

The results are displayed even if the V-(D)-J-REGION (V-DOMAIN) are partial and/or not fully characterized, the only requirement being that at least the V-REGION has been identified, which is the condition for IMGT/V-QUEST to give results.

### IMGT/V-QUEST Detailed view results for scFv

**Fig. 4:**
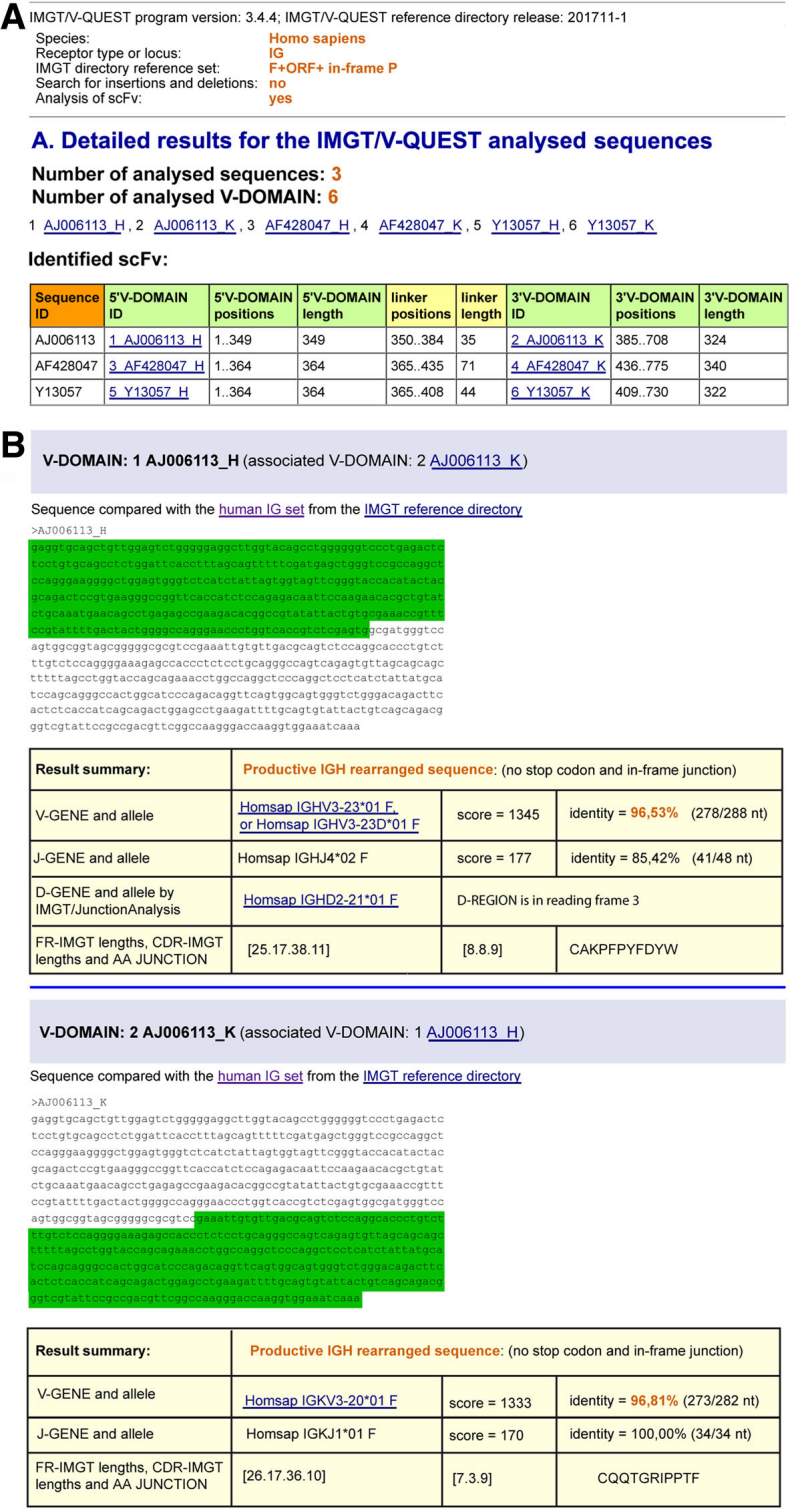
IMGT/V-QUEST Detailed view results for IG scFv sequences. **a** The top of the Detailed view results recalls the parameters for the analysis and provides the number of analyzed sequences (here, 3) and the number of analyzed V-DOMAIN (here, 6). The “Identified scFv” table indicates, for each identified scFv in the submitted sequence set, the positions and length of the 5′V-DOMAIN, linker and 3′V-DOMAIN in the ‘V-orientated’ scFv. Clicking on the 5′V-DOMAIN ID or 3′V-DOMAIN ID leads to the corresponding detailed analysis. **b** Sequence and Result summary for the two V-(D)-J-REGION (V-DOMAIN) of a scFv are shown. The part of the scFv FASTA sequence colored in green corresponds to the analyzed V-DOMAIN. AJ006113, AF428047, Y13057 are accession numbers in the IMGT/LIGM-DB database [21]. Other detailed results for each V-DOMAIN comprise 14 displays (not shown) as listed in Detailed view

The top of the page IMGT/V-QUEST Detailed view results for scFv (Fig. 4a) recalls the IMGT/V-QUEST program version, IMGT/V-QUEST reference directory release, and then the selected parameters: Species, Receptor type or locus (IG or TR), IMGT reference directory set (e.g., F + ORF + in-frame P), and options ‘Search for insertions and deletions’ (yes or no) and, for the current purpose, ‘Analysis of scFv’ (yes).

The Detailed results show the Number of analysed sequences (here, 3) and the Number of analysed V-DOMAIN (here, 6). The table “Identified scFv” comprises one line per identified scFv in the submitted sequence set. It includes the sequence ID (the one from the flat file header), the 5′V-DOMAIN ID, positions and length, the linker positions and length, the 3′V-DOMAIN ID, positions and length (Fig. 4a).

The 5′V-DOMAIN ID and 3′V-DOMAIN ID consists of the sequence ID, followed by an underscore and a capital letter for the locus as identified by IMGT/V-QUEST (H, K, L for IGH, IGK and IGL, and A, B, D and G for TRA, TRB, TRD and TRG, respectively), and preceded by a number which indicates the V domain analysis order in the submitted set. Clicking on the 5′V-DOMAIN ID or 3′V-DOMAIN ID link leads to the corresponding classical detailed view (Fig. 4b).

The complete V-orientated sequence of the scFv is shown in the result of each domain, with the corresponding analysed V domain being highlighted in green.

If the option ‘Search for insertions and deletions’ has been selected, insertions detected are reported in capital letters in the sequence of the corresponding V domain(s). The IMGT/V-QUEST results per domain are given after filling the deletions and removing the insertions [25].

The Result summary table of each V domain (Fig. 4b) is followed by the 14 classical displays of Detailed view results (not shown) [32].

### IMGT/V-QUEST Synthesis view results for scFv

**Fig. 5:**
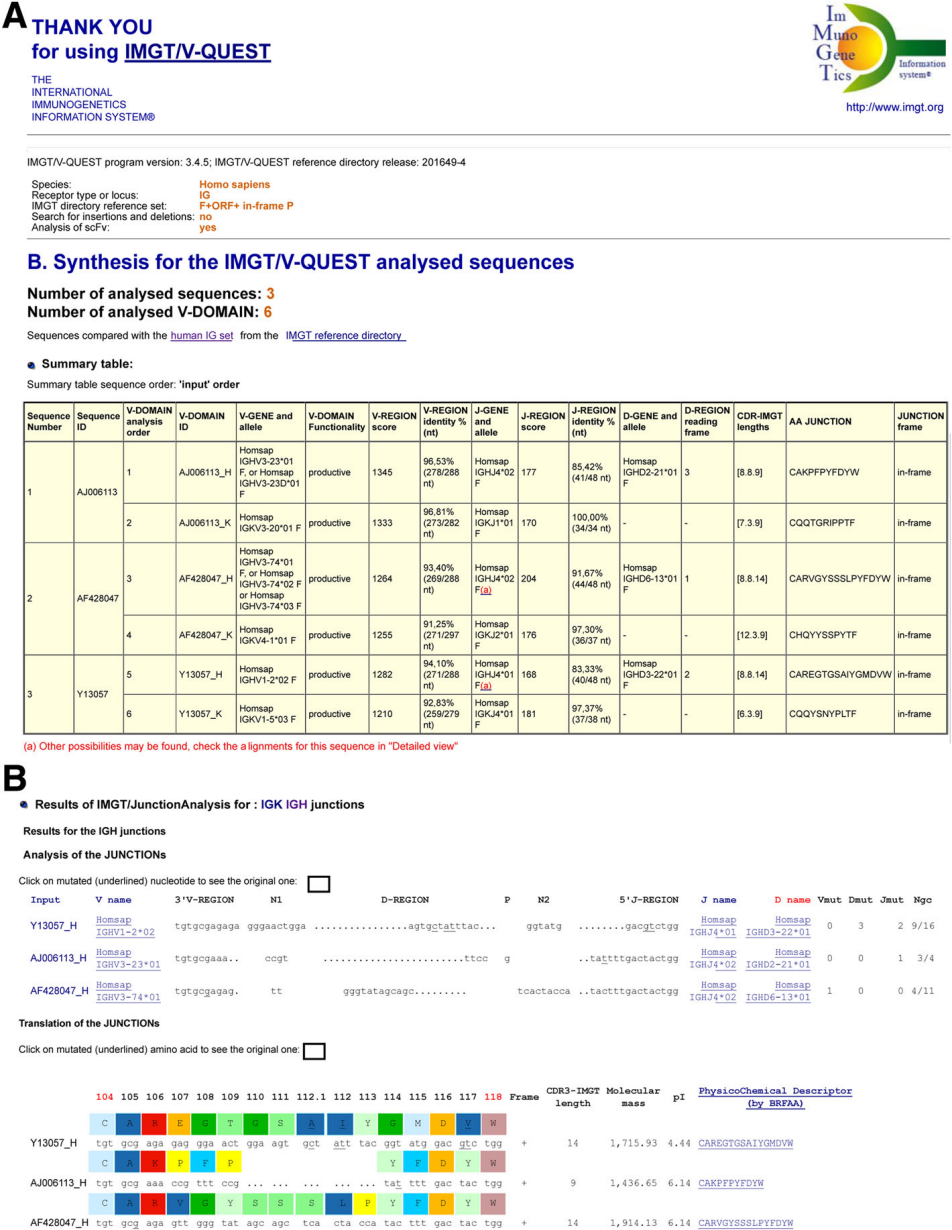
IMGT/V-QUEST Synthesis view results for IG scFv sequences. **a** Three scFv were analyzed with the option “Analysis of single chain Fragment variable (scFv)”. The top of the Synthesis view recalls the parameters for the analysis and provides the number of analyzed sequences (here, 3) and the number of analyzed V-DOMAIN (here, 6). The Summary table includes one line per V-DOMAIN and 2 lines per identified scFv in the submitted sequence set, identified by their order number in the set and their sequence ID. The order of V-DOMAIN analysis is indicated on the left of the V-DOMAIN ID. AJ006113, AF428047, Y13057 are accession numbers in the IMGT/LIGM-DB database [21]. Other results for each V-DOMAIN comprise 8 displays (not shown) as listed in Synthesis view. **b** Results of IMGT/JunctionAnalysis for the VH domain of the 3 scFv

The top of the page IMGT/V-QUEST Synthesis view results for scFv (Fig. 5) recalls, as for the Detailed view results above, the IMGT/V-QUEST program version, IMGT/V-QUEST reference directory release, and then the selected parameters: Species, Receptor type or locus (IG or TR), IMGT reference directory set (e.g., F + ORF + in-frame P, and options ‘Search for insertions and deletions’ (yes or no) and, for the current purpose, ‘Analysis of scFv’ (yes).

Each identified V-(D)-J-REGION (V-DOMAIN) appears individually on a different line of the Summary table. Pairs of V-DOMAIN belonging to the same scFv, are identified by having the same sequence ID (Fig. 5). For each identified V-(D)-J-REGION (V-DOMAIN), the classical results are displayed (V-GENE and allele, V-DOMAIN Functionality, V-REGION score, V-REGION identity % (nt), J-GENE and allele, J-REGION score, J-REGION identity % (nt), D-GENE and allele, D-REGION reading frame, CDR-IMGT lengths, AA JUNCTION, JUNCTION frame). Below the Summary table, Results of IMGT/JunctionAnalysis (comparison of JUNCTION of V-DOMAIN belonging to the same locus, e.g., IGH in Fig. 5) and Alignment with the closest alleles (comparison of V-DOMAIN expressing the same V gene and allele) are provided for an analysis between V-DOMAIN of different scFv.

### IMGT/V-QUEST Excel file or IMGT/HighV-QUEST CSV files results for scFv

Classically, the IMGT/V-QUEST Excel file or, for NGS, the IMGT/HighV-QUEST comma separated values (CSV) files results include eleven Excel spreadsheets or CSV files, respectively [35]. Typically, the first ten Excel spreadsheets and CSV files include one line per identified and analyzed V-(D)-J-REGION (V-DOMAIN) (for scFv, there are therefore two lines corresponding to the two V domains for each scFv). The file 11_Parameters indicates the number of submitted scFv sequences and the number of analyzed V-DOMAIN.

**Fig. 6:**
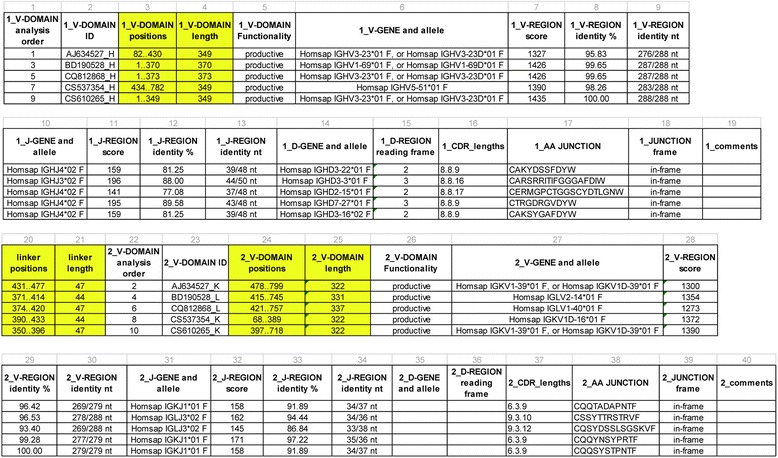
IMGT/V-QUEST 12_scFv spreadsheet of the Excel file. Only available for the option “Analysis of single chain Fragment variable (scFv)”). It includes one line per identified scFv in the submitted sequence set with the characterization of the two V-DOMAIN by 19 fields each prefixed by “1” or “2”. In order to facilitate data extraction, VH (for IG), V-BETA or V-DELTA (for TR) domains are always displayed from column 1 to 19 (prefixed by “1”) and VL (for IG), V-ALPHA or V-GAMMA (for TR) are always displayed from column 22 to 40 (prefixed by “2”) (with exception for scFv composed of 2 VH or of 2 VL domains). Columns 20 and 21 correspond to linker positions and linker length, respectively. Yellow columns have been specifically created for scFv analysis. 1_V-DOMAIN analysis order and 2_V-DOMAIN analysis order columns indicate the V-DOMAIN analysis order which is also reported in the 10 classical spreadsheets of Excel file in IMGT/V-QUEST and in the CSV files in IMGT/HighV-QUEST

An additional file “12_scFv” (Fig. 6) is specific to the scFv analysis and is automatically included in the results if the option “Analysis of single chain Fragment variable (scFv)” has been selected. The “12_scFv” file includes a single line per submitted sequence identified as an scFv (i.e., with two V-DOMAIN, or at least two V-REGION, identified in the same sequence). Each line crosses two sets of 19 columns, prefixed by “1_” and by “2_”, respectively, which correspond to the results of the two V domains of the scFv, with between them, two columns for the ‘linker positions’ and the ‘linker length’ in the V-orientated scFv sequence. It should be noted that the assignment “1_” and “2_” in this file is arbitrary (it is independent on the V domain analysis order number and on the relative positions of the V domains in the V-orientated scFv). In order to facilitate data extraction and reuse, VH, V-BETA and V-DELTA are in the “1_” column set for scFv which contain paired VH-VL (V-KAPPA or V-LAMBDA), V-ALPHA-V-BETA or V-GAMMA-V-DELTA (Table 2).

**Table 2:** Associations of scFv V-DOMAIN analyzed by IMGT/V-QUEST

IG scFv		TR scFv	
5′V-DOMAIN	3′V-DOMAIN	5′V-DOMAIN	3′V-DOMAIN
VH	V-KAPPA	V-ALPHA	V-BETA
VH	V-LAMBDA	V-BETA	V-ALPHA
VH	VH	V-GAMMA	V-DELTA
V-KAPPA	VH	V-DELTA	V-GAMMA
V-KAPPA	V-LAMBDA		
V-KAPPA	V-KAPPA		
V-LAMBDA	VH		
V-LAMBDA	V-KAPPA		
V-LAMBDA	V-LAMBDA		

The IG scFv are usually made of a VH and of VL. The order of the V-DOMAIN in an scFv can be either VH-linker-VL or VL-linker-VH. Both V domains of an scFv are transcribed in a single chain and are necessarily in the same orientation of transcription. Different associations of V domains (the two domains being in the same orientation) are possible for the IG or for the TR. Analysis of scFv V-DOMAIN by IMGT/V-QUEST is done against a single species. For the TR, other V domain associations per permutation between any two V domains (not shown) are also analyzable.

Each set of 19 columns for each V domain comprises the following fields:

1. V-DOMAIN analysis order: order in the submitted scFv sequences set (as in the ten first spreadsheets or files),
2. V-DOMAIN ID: V-DOMAIN identifier (SequenceName_LocusLetter),
3. V-DOMAIN positions: begin and end position of the identified V-DOMAIN in the V-orientated scFv sequence,
4. V-DOMAIN length: as determined by the begin and end positions,
5. V-DOMAIN Functionality: productive or unproductive,
6. V-GENE and allele: IMGT gene and allele name of the closest germline V-REGION,
7. V-REGION score: alignment score with the closest germline V-REGION,
8. V-REGION identity %: identity percentage with the closest germline V-REGION,
9. V-REGION identity nt: number of identical nt with the closest germline V-REGION,
10. J-GENE and allele: IMGT gene and allele name of the closest germline J-REGION,
11. J-REGION score: alignment score with the closest germline J-REGION,
12. J-REGION identity %: identity percentage with the closest germline J-REGION,
13. J-REGION identity nt: number of identical nt with the closest V germline J-REGION,
14. D-GENE and allele: IMGT gene and allele name of the closest germline D-REGION (as identified by IMGT/JunctionAnalysis),
15. D-REGION reading frame: reading frame 1, 2 or 3 (as identified by IMGT/JunctionAnalysis),
16. CDR_lengths: length of the 3 CDR-IMGT,
17. AA JUNCTION: amino acid sequence of the junction,
18. JUNCTION frame: frame of the junction (in-frame or out-of-frame),
19. Comments: to highlight the particularities of the V-DOMAIN, if any.

It should be noted that sequences not identified as scFv (i.e., for which only a single (or no) V-DOMAIN or V-REGION is identified) are not integrated in the “12_scFv” spreadsheet or file, so this spreadsheet or file may be empty if none of the submitted sequences are identified as scFv.

As the online version of IMGT/V-QUEST can analyze 50 sequences per run, the results for scFv analysis may potentially include the analysis of up to 100 V-DOMAIN. With the option “Search for insertions and deletions”, the number of submitted sequences is restricted to 10, and the results for scFv may include the analysis of up to 20 V-DOMAIN.

In the IMGT/HighV-QUEST, the option “Search for insertions and deletions” is selected by default and the analysis includes all the identified V-DOMAIN. The new advanced functionality “Analysis of single chain Fragment variable (scFv)” provides the identification and characterization of, theoretically, up to one million domains for 500,000 submitted scFv sequences. This functionality has introduced, for the first time, the possibility of analysing simultaneously the two V domains of large scFv data sets from combinatorial libraries.

## Discussion

In antibodies and T cell receptors, the antigen binding sites comprise two V-DOMAIN which are paired at the N-terminal end of the heavy and light chains for the IG and of the alpha and beta (or gamma and delta) chains for the TR [1–3]. The pairing of the two V-DOMAIN is reproduced in scFv in which the two V-DOMAIN are connected by a peptide linker. These engineered monovalent molecules were first expressed in *Escherichia coli* [43, 44] and then at the surface of filamentous phages. This methodology combined with the polymerase chain reaction (PCR) amplification of variable domains was the starting point of the construction of scFv phage combinatorial libraries [45–47], by-passing hybridoma technology and animal antibody humanization. The scFv can be expressed in various systems (bacteria, phages, yeast, plant, mammalian cells), leading to the generation of many different scFv combinatorial libraries and to the development of various technologies (such as phage or ribosome display) as an efficient tool for the screening, selection and enrichment of antibodies with a given specificity. The selection from scFv combinatorial libraries is widely used for the discovery of novel antibody specificities for diagnostic and therapy [48–51].

Next generation sequencing (NGS) has recently emerged as a new method for the high-throughput characterization of IG and TR immune repertoires both in vivo and in vitro. Currently available NGS platforms allow the simultaneous sequencing of millions of reads. However, two challenges remain for the NGS sequencing of scFv: first, the scFv length is > 800 bp, which is too long for most NGS platforms; and second, there is no tool for the analysis of two V domains in a single chain. Up to now, NGS methods have only provided reads encompassing one V domain (400 bp), therefore losing a critical piece of information found in scFv sequences, that of the association of two specific V domains (VH and VL for the IG) by the peptide linker. Although a few approaches have been proposed, retrieving information regarding V domain association has still not been solved [52–54].

As reliable data depend on high-quality and long enough sequences to contain the full-length scFv, the new functionality “Analysis of single chain Fragment variable (scFv)” was implemented for providing the identification and full characterization of the two V domains in scFv sequences or NGS reads fulfilling these criteria.

## Conclusions

The functionality “Analysis of single chain Fragment variable (scFv)” provides the identification and full characterization of the two V domains of full-length scFv in IMGT/V-QUEST online or, for NGS, in IMGT/HighV-QUEST. The functionality was used to analyse >450,000 reads of about 1000 bp, obtained from a combinatorial library, generated with the Pacific Biosciences (PacBio) RS II platform using single molecule, real-time (SMRT) circular consensus sequencing (CCS). The two V domains were identified and characterized in all reads of high-quality and sufficient length. The “Analysis of single chain Fragment variable (scFv)” will facilitate and improve the description of the scFv content of combinatorial libraries, a key information in therapeutic antibody discovery, selection and development.

The need for the analysis of sequences containing two V domains from expressed repertoires is also rapidly rising. NGS single-cell sequencing of paired chains have been obtained by a technology comprising flow focusing and encapsulation of single cells in emulsions containing magnetic beads for mRNA capture, reverse-transcription of mRNA transcripts, physical linkage of the partners by overlap extension PCR, and NGS sequencing [55]. Other developments of paired IG and TR sequences include paired recovery of transcripts and concatenation per single cell [56], single cell paired sequencing [57], capture strategies [58]. IMGT/V-QUEST and IMGT/HighV-QUEST perform classically on sequences of paired chains identified by bar-coding of single cells, each chain having a single V-DOMAN. In contrast, if the sequences of the paired chains are physically linked, the functionality “Analysis of single chain Fragment variable (scFv)” should be selected in order to identify and describe the two V-DOMAIN. Indeed, this functionality for scFv sequence analysis is generic for IG and TR and can be used without modification for libraries of single B or T cell concatenated paired expressed chains, and will facilitate the identification of novel paratopes in infections, cancers, autoimmune diseases or neurodegenerative diseases.

## Abbreviations

2D: Two-dimensional
3D: Three-dimensional
AA: Amino acid
bp: Base pair
C: Constant
CCS: Circular consensus sequencing
CDR: Complementarity determining region
CPCA: Composite protein for clinical applications
CSV: Comma separated values
D: Diversity
Da: Dalton
F: Functional
FPIA: Fusion protein for immune applications
FR: Framework region
H: Heavy
ID: Identifier
IG: Immunoglobulin
IgSF: Immunoglobulin superfamily
J: Joining
L: Light
MH: Major histocompatibility
MhSF: Major histocompatibility superfamily
NGS: Next generation sequencing
nt: Nucleotide
ORF: Open reading frame
P: Pseudogene
RPI: Related protein of the immune system
scFv: Single chain Fragment variable
SMRT: Single molecule, real-time
TR: T cell receptor
V: Variable
VH: Variable heavy
VL: Variable light
